# Supplementary material for: MSBooster: improving peptide identification rates using deep learning-based features
Source: Nat Commun. 2023 Jul 27;14:4539. doi: 10.1038/s41467-023-40129-9 (PMC10374903; doi:10.1038/s41467-023-40129-9)
Supplement: Supplementary file 1 — Supplementary Information [file 41467_2023_40129_MOESM1_ESM.pdf]

Supplement to: "MSBooster: Improving Peptide Identification Rates using Deep Learning-Based Features"

Kevin L Yang[1], Fengchao Yu[2]*, Guo Ci Teo[2], Kai Li[1], Vadim Demichev[3,4], Markus Ralser[3,5,6], Alexey I Nesvizhskii[1,2]*

[1]Department of Computational Medicine and Bioinformatics, University of Michigan, Ann Arbor, MI, USA

[2]Department of Pathology, University of Michigan, Ann Arbor, MI, USA

[3]Department of Biochemistry, Charité Universitätsmedizin, Berlin, Germany

[4]Department of Biochemistry, University of Cambridge, Cambridge, UK

[5]The Wellcome Centre for Human Genetics, Nuffield Department of Medicine, University of Oxford, UK

[6]Max Planck Institute for Molecular Genetics, Berlin, Germany

*Correspondence to F.Y. (yufe@umich.edu) and A.I.N. (nesvi@med.umich.edu)

Contents

Supplementary Figure 1……………………………………………………………………………3
Supplementary Figure 2……………………………………………………………………………4
Supplementary Figure 3……………………………………………………………………………5
Supplementary Figure 4……………………………………………………………………………6
Supplementary Figure 5……………………………………………………………………………7
Supplementary Figure 6……………………………………………………………………………8
Supplementary Figure 7……………………………………………………………………………9
Supplementary Figure 8…………………………………………………………………………..10
Supplementary Figure 9…………………………………………………………………………..11
Supplementary Figure 10…………………………………………………………………………12
Supplementary Figure 11…………………………………………………………………………13
Supplementary Figure 12…………………………………………………………………………14
Supplementary Figure 13…………………………………………………………………………15
Supplementary Note 1……………………………………………………………………………16
Supplementary Note 2……………………………………………………………………………17
References…………………………………………………………………………………………18

Supplementary Figures

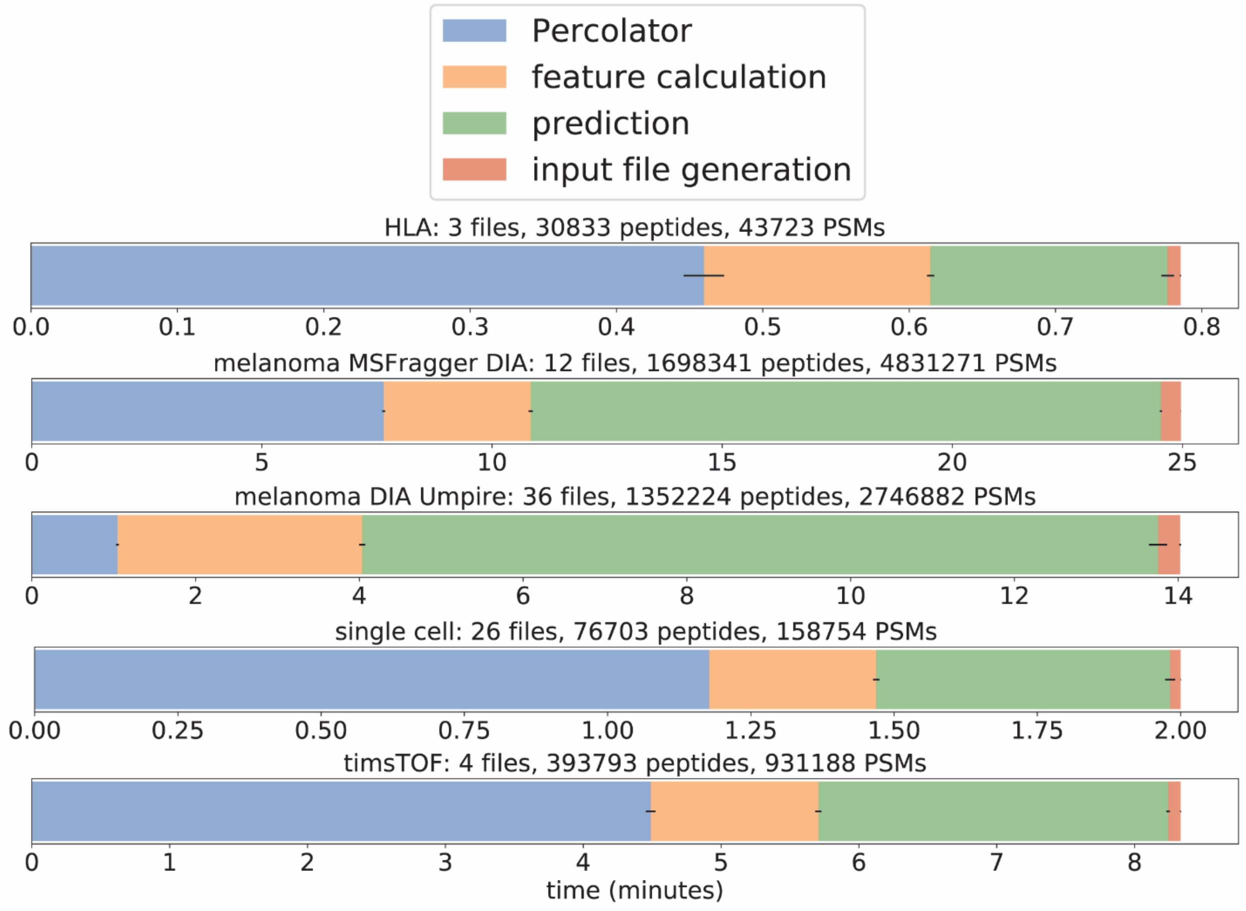

Supplementary Figure 1. Timing of MSBooster steps and Percolator rescoring. Error bars show the standard deviation from running the software ten times. For each subfigure, the dataset, the number of mzml/pin file pairs, the number of unique peptides in the pin files, and the total number of PSMs are listed. Data are presented as mean values +/- the standard deviation. Source data are provided as a Source Data file.

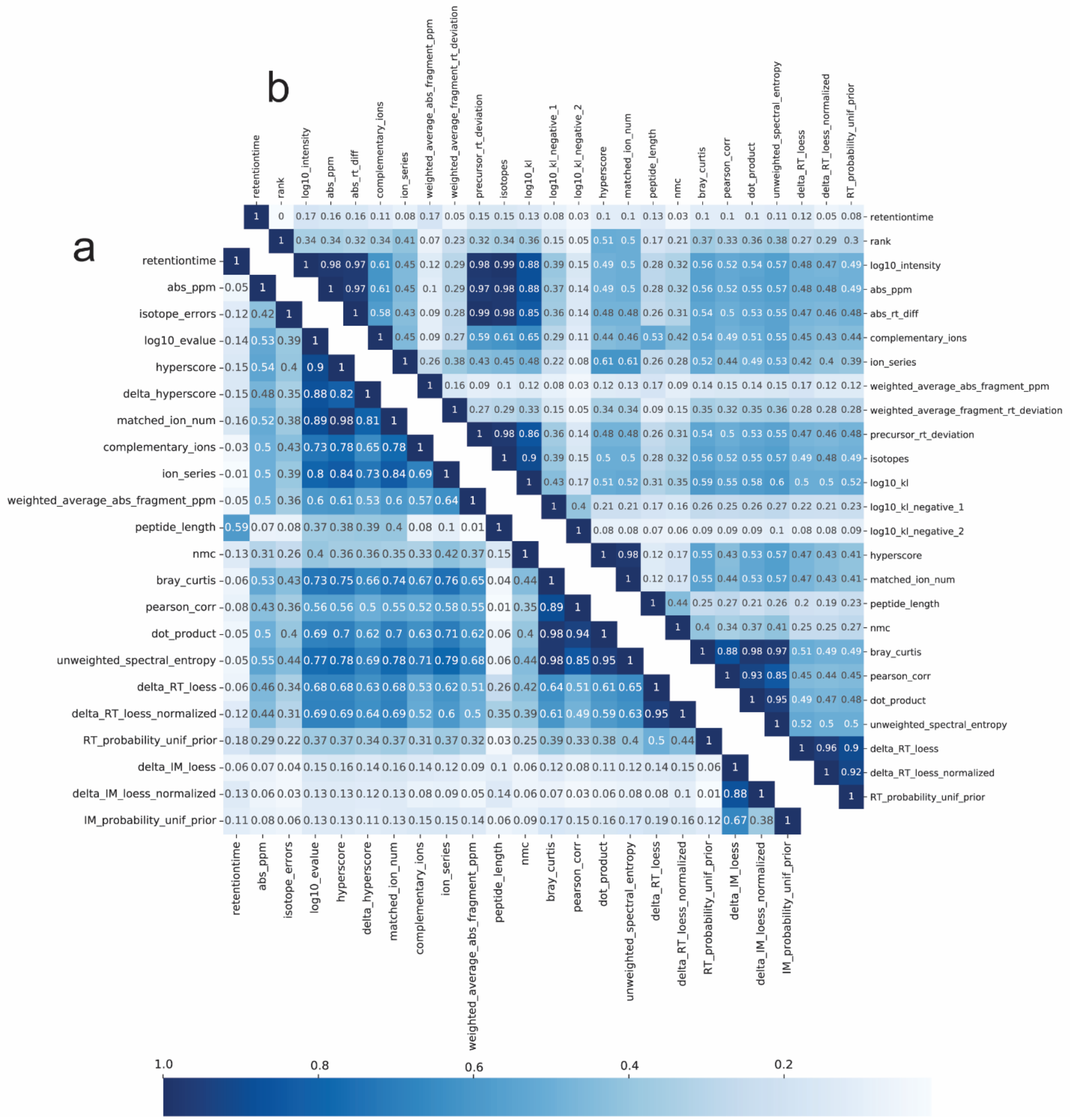

Supplementary Figure 2. Feature correlations. Spearman's correlation was calculated between MSBooster features and all features reported by MSFragger (a) and MSFragger-DIA (b). (a) was produced from one timsTOF file, (b) from one melanoma file. Source data are provided as a Source Data file.

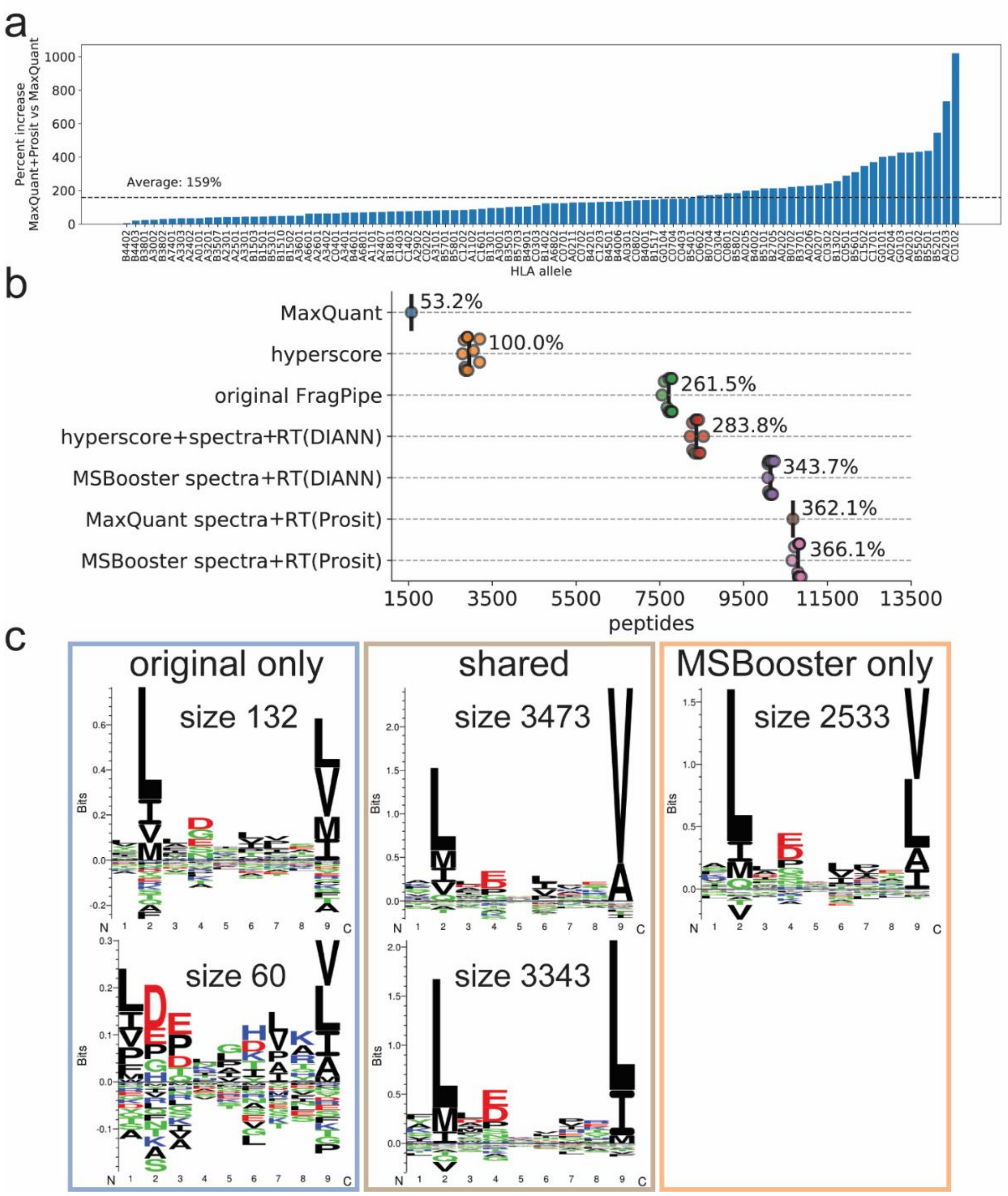

Supplementary Figure 3. HLA immunopeptidome PSM rescoring. (a) The percent increase in peptides identified when rescoring MaxQuant results with Prosit. Each bar represents the results for a different monoallelic cell line from Sarkizova et al., 2020. (b) Swarmplot of the number of HLA peptides reported in comparison to only using hyperscore or in comparison to MaxQuant rescoring with Prosit. “Original FragPipe” indicates results without using deep learning features, only using hyperscore and other features calculated by MSFragger. “MSBooster spectra+RT (DIANN)” has the MSBooster generated features added on and is equivalent to “spectra+RT” in Fig 2a. (c) GibbsCluster-generated motifs assigned to each peptide subset from the Venn diagram in Fig 2b. Source data are provided as a Source Data file.

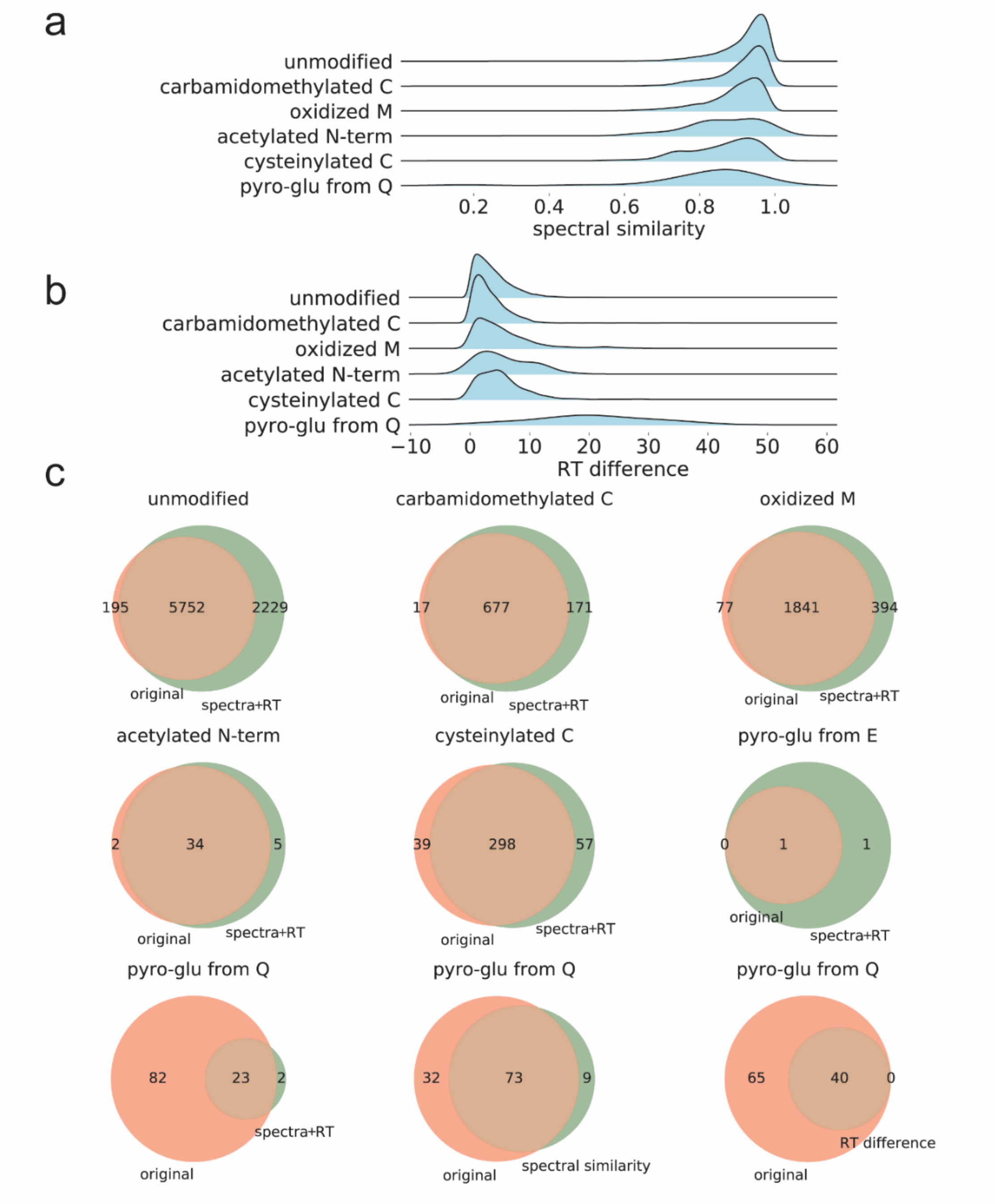

Supplementary Figure 4. Score distributions for unmodified and modified HLA peptides. (a) Spectral similarity values for accepted PSMs at PSM FDR < 1% and peptide FDR < 1%. (b) The same as (a) but for RT difference values. Note that these values are not log-normalized as in other figures. Pyro-glutamation on E was included in the search, but only one was detected and therefore excluded from visualization. (c) Venn diagram of the number of peptides with each modification before and after MSBooster rescoring. The bottom row is for pyroglutamation from Q, either rescored with spectral similarity, RT difference, or both together (spectra+RT). Source data are provided as a Source Data file.

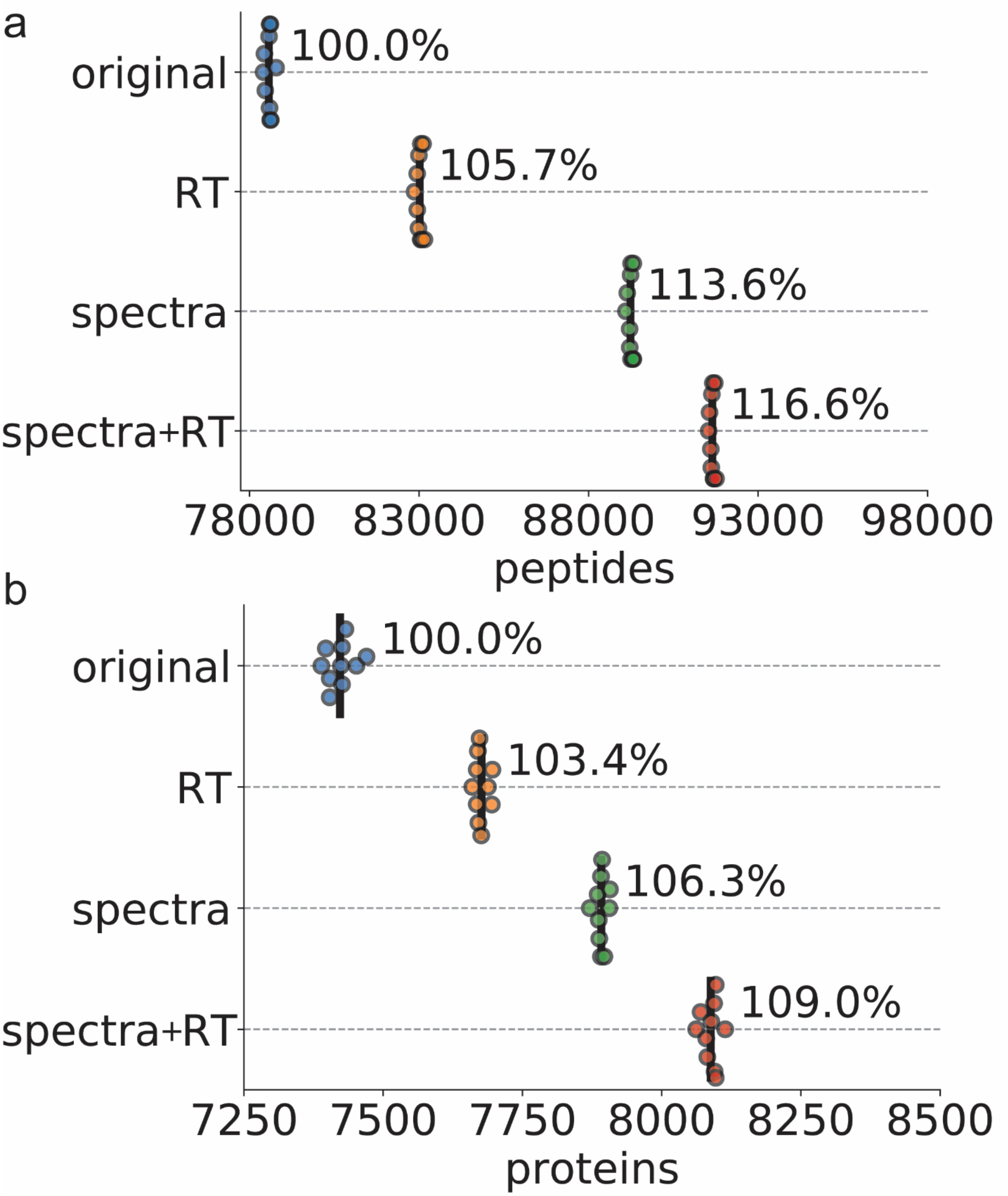

Supplementary Figure 5. Melanoma DIA rescoring with DIA-Umpire. Swarmplots of the number of peptides (a) or proteins (b) reported at 1% FDR. Source data are provided as a Source Data file.

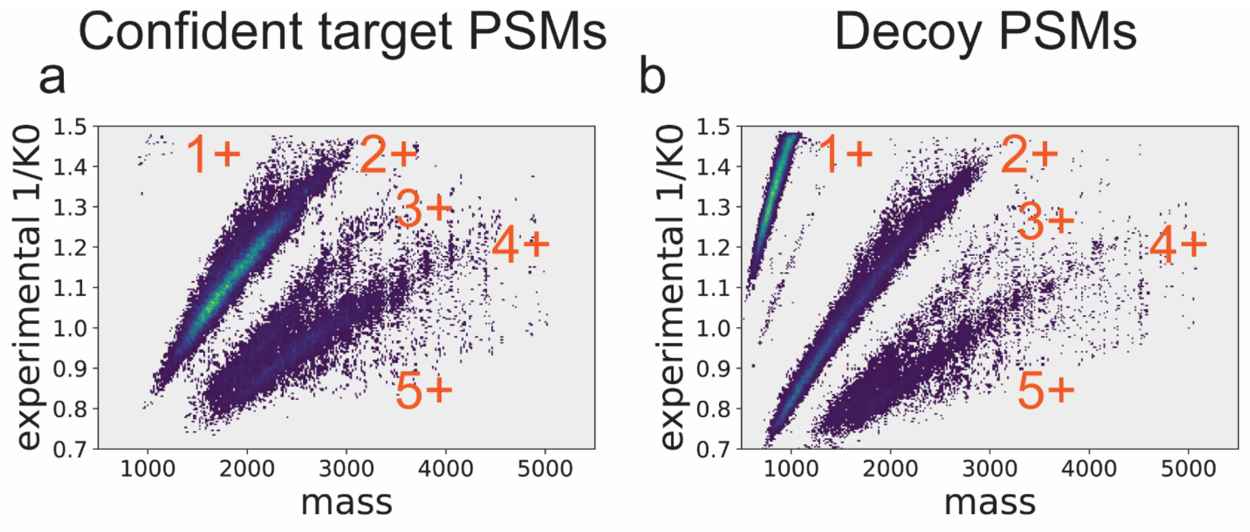

Supplementary Figure 6. Inverse ion mobility dependence 1/K0 on mass and charge. (a-b) Scatter density plots are shown for (a) confident target PSMs and (b) decoy PSMs, where brighter colors represent higher density. Figures a-b were generated using data from a single DDA PASEF run. Source data are provided as a Source Data file.

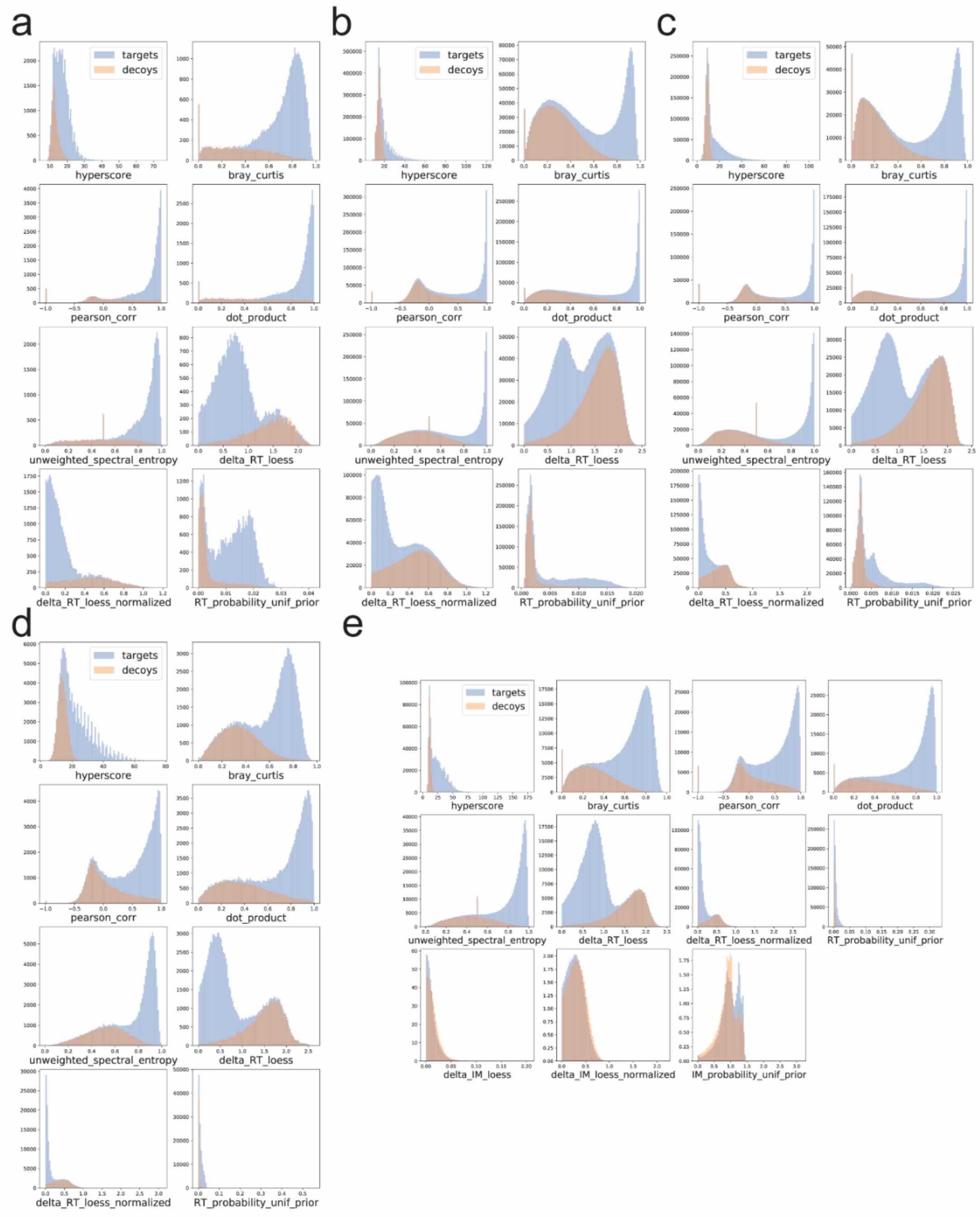

Supplementary Figure 7. PSM score distributions for MSBooster features. Target PSMs are in blue, decoy PSMs in orange. The datasets analyzed were HLA (a), melanoma processed by MSFragger DIA (b), melanoma processed by DIA-Umpire with MSFragger (c), single cell nanoPOTS (d), and timsTOF (e). RT and IM features were log-transformed to better visualize target-decoy separation using the conversion log10(value + 1). Spectral and RT figures have

counts on the y-axis, IM has density. Targets are depicted in blue, decoys in orange. Source data are provided as a Source Data file.

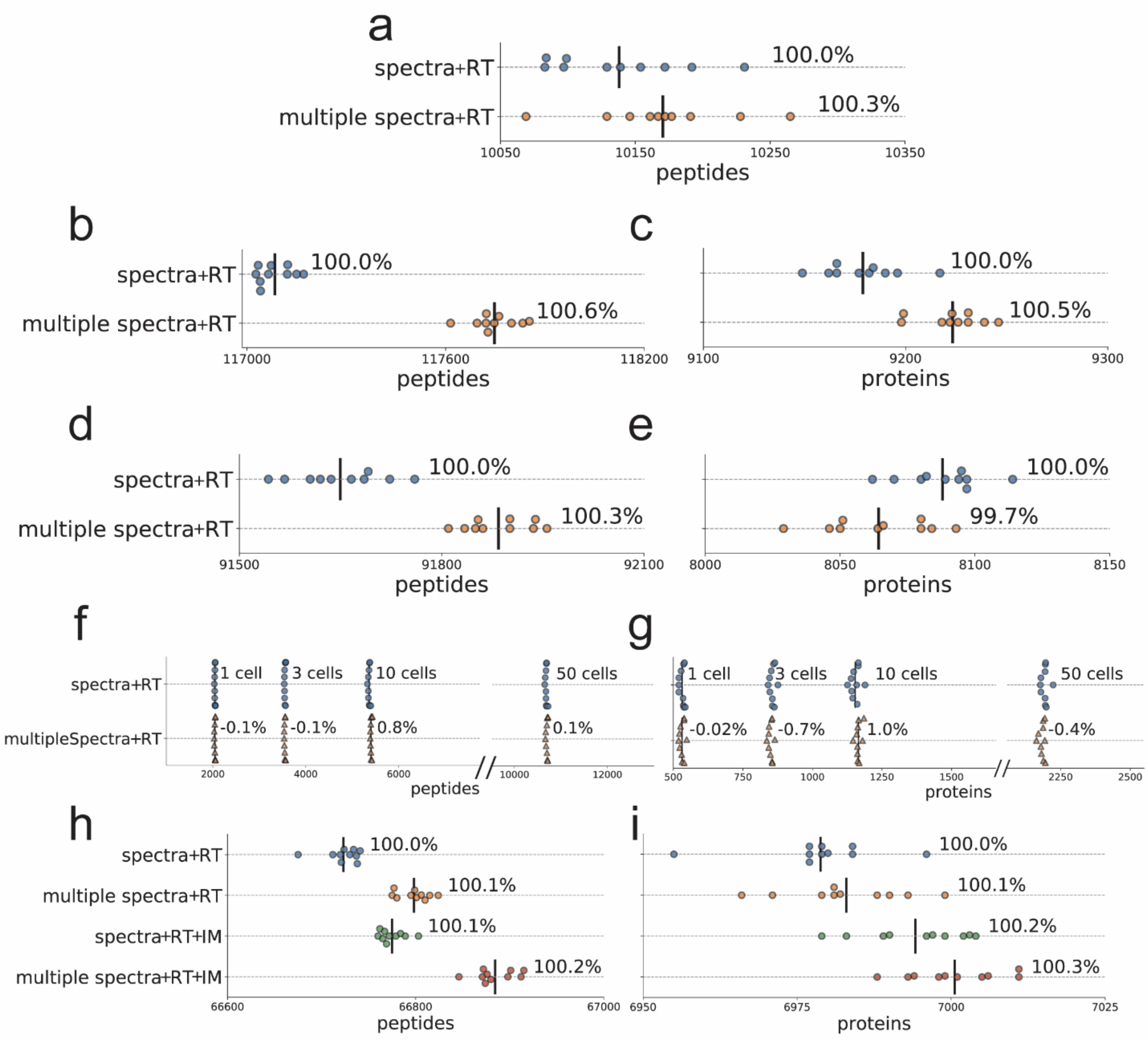

Supplementary Figure 8. Utility of multiple correlated features. Single features (unweighted spectral entropy + delta RT LOESS = spectra+RT; unweighted spectral entropy + delta RT LOESS + IM probability uniform prior = spectra+RT+IM) are compared against multiple features (everything listed in Supplementary Note 1) for Percolator rescoring. Significance was calculated using two-sided independent t-tests with p<0.01, and p-values are listed in Supplementary Data 1. The numbers reported are for HLA peptides (a), melanoma peptides (b) and proteins (c) with MSFragger-DIA, melanoma peptides (d) and proteins (e) with DIA-Umpire and MSFragger, single cell peptides (f) and proteins (g), and timsTOF peptides (h) and proteins (i). Source data are provided as a Source Data file.

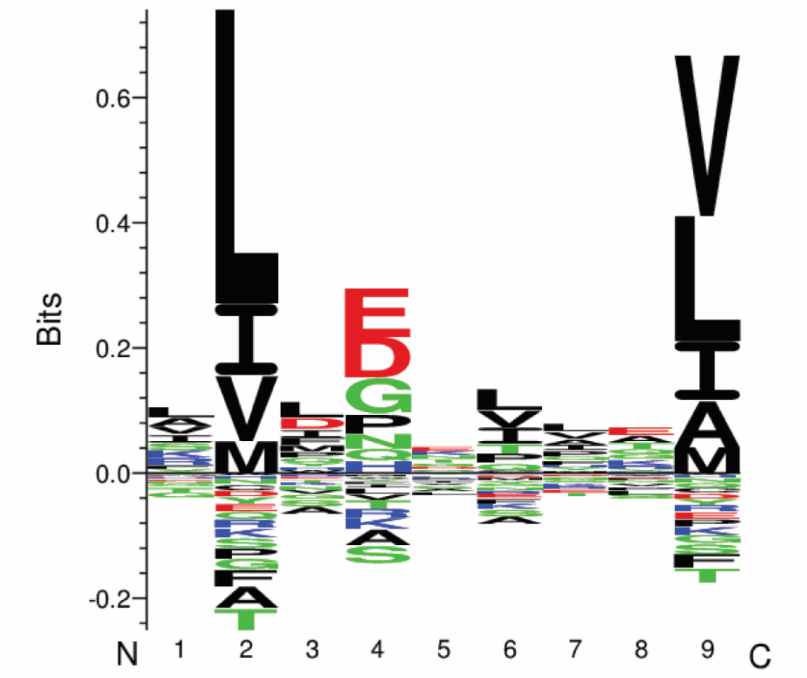

Supplementary Figure 9. HLA motif for new peptides from rescoring the HLA dataset with multiple correlated features.

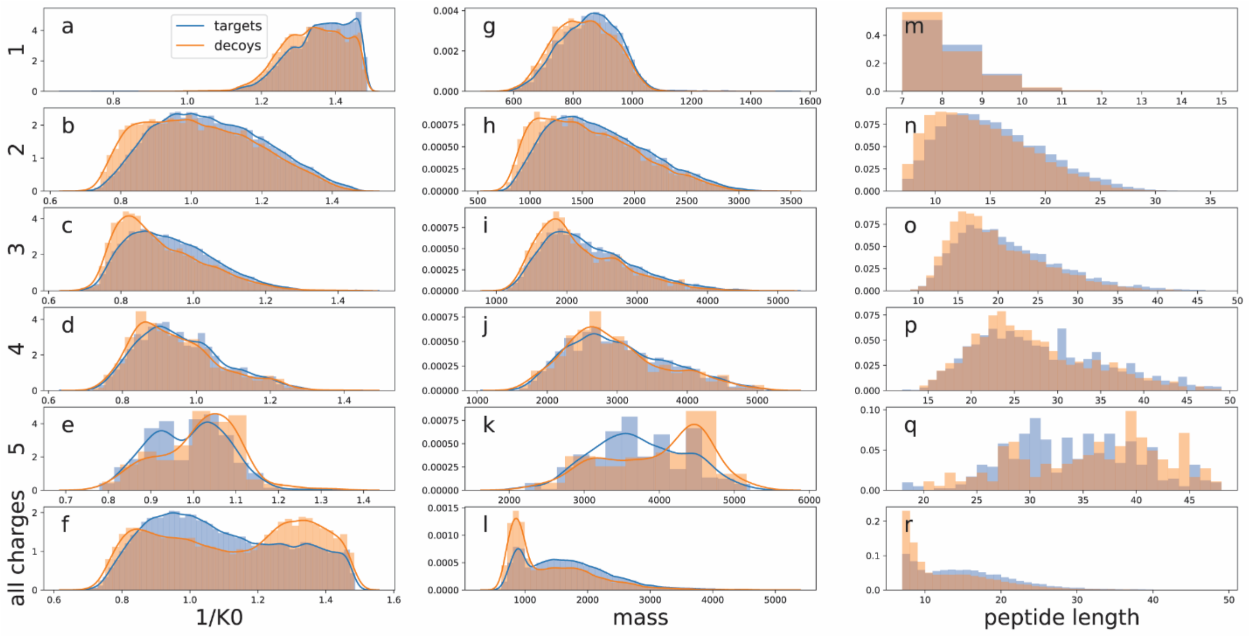

Supplementary Figure 10. Target-decoy PSM distributions for different charge states and features. Target-decoy distributions for inverse ion mobility (a-f), mass (g-l), and peptide length (m-r) are shown for precursors of charge 1-5 or all charges together. All subfigures show relative density rather than PSM counts to better show target-decoy separation. Targets are depicted in blue, decoys in orange. Source data are provided as a Source Data file.

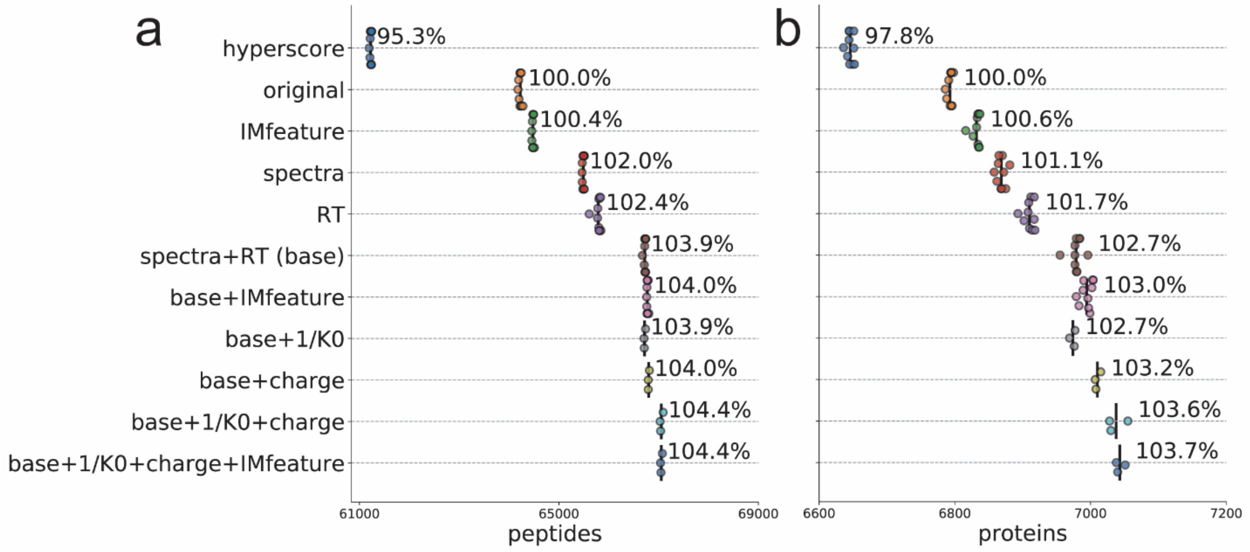

Supplementary Figure 11. Swarmplot of peptides (a) and proteins (b) reported at 1% FDR for the timsTOF Hela dataset. The “IMfeature” used was IM probability uniform prior. 1/K0 is the experimental inverse ion mobility. Charge was one-hot encoded between charges 1 and 7. “Base” represents the default in FragPipe with only spectral and RT similarity features. Source data are provided as a Source Data file.

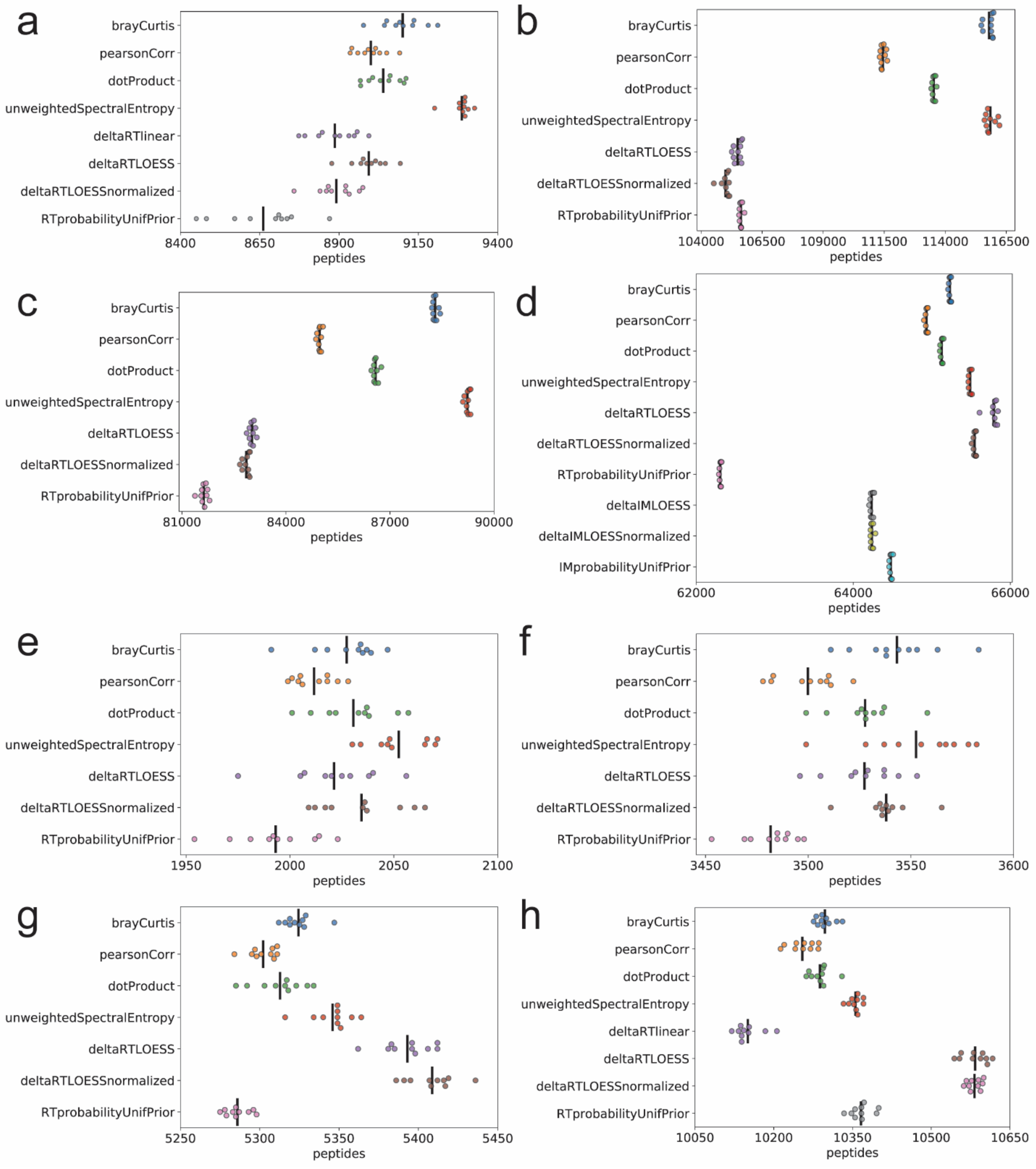

Supplementary Figure 12. Peptides reported when using different MSBooster features. Swarmplots for HLA (a), melanoma MSFragger DIA (b), melanoma DIAU (c), timsTOF (d), 1 cell (e), 3 cells (f), 10 cells (g), 50 cells (h). Each dataset was processed 10 times by Percolator, each time with a different random seed 1-10. The black line indicates mean peptides reported over the 10 runs. Source data are provided as a Source Data file.

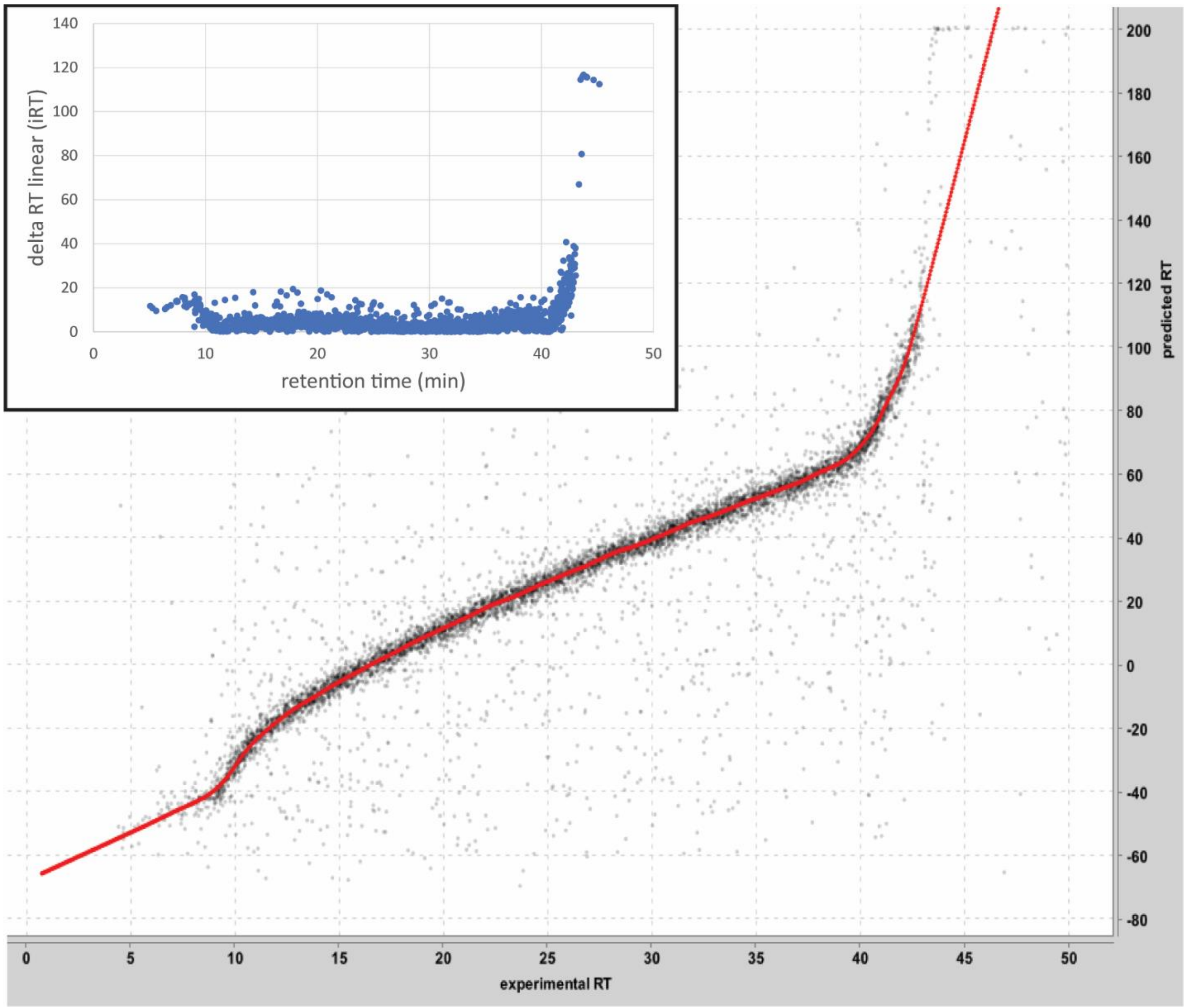

Supplementary Figure 13. A plotted calibration curve between the experimental and predicted RT scales. The data shown here is from one replicate of a 50-cell run from the nanoPOTS data (Williams et al.). The red line represents the values estimated by LOESS regression. Individual black dots are those PSMs with spectral similarity greater than 0 and log(expectation value) less than a user-defined threshold, which is by default -3.5. The inset shows the relationship between the experimental RT and difference between predicted and experimental RT when using only a linear regression for calibration (delta RT linear). Source data are provided as a Source Data file.

Supplementary Notes

Supplementary Notes 1. Features

Available features in MSBooster are listed below, along with an equation and source for less commonly used metrics. By default in FragPipe, the unweighted spectral entropy and delta RT LOESS only are used.

- Spectral similarity: Unweighted spectral entropy requires fragment ion intensity vectors to sum to 1. Other metrics require unit normalization (i.e. the sum of squared intensities equals 1). *p* stands for predicted fragment, *P* for predicted intensity vector, *m* for matched experimental fragment, and *M* for matched experimental intensity vector. By default, the top 12 highest predicted intensity fragments are used.
  - Bray Curtis[1]: $1 - \frac{\Sigma_{i=1}^{n}|p_i - m_i|}{\Sigma_{i=1}^{n} p_i + m_i}$
  - Pearson's correlation: If no matched experimental fragment ions, value is -1
  - Dot Product
  - Unweighted spectral entropy[2]: $1 - \frac{2S_{PM} - S_P - S_M}{ln\,4}$, where entropy *S* = $-\sum_{i=1}^{n} f_i \, ln\, f_i$, and S_{PM} is the sum of predicted and matched vectors S_P and S_M divided by 2
- Retention time similarity
  - delta RT loess: Described in Methods (Retention time and ion mobility calibration).

    - delta RT loess normalized: Similar to delta RT loess but divided by the interquartile range of predicted RTs for high confidence PSMs in the experimental RT vicinity.
    - RT probability Unif Prior: Described in Methods (Kernel density estimation of predicted retention time and ion mobility distributions).
- Ion mobility similarity
    - Features are like those for RT but done separately for each charge. Only charges +1-7 are supported.

Supplementary Notes 2. IM feature testing

Because our IM features only marginally improved identifications, we further explored this data to see if we could improve our features. We noted a higher density of decoy PSMs at lower 1/K0 values (around 0.7-0.8) compared to confident target PSMs (**Fig 6c-d, Supplementary Fig 10**). Similarly, decoy PSMs tend to have lower masses and shorter lengths than target PSMs for lower charge states. We hypothesized that adding these simpler features may be beneficial for performance. Peptide mass and length have already been incorporated as features in the MSFragger-generated pin file. We tested adding one-hot-encoded charge and 1/K0 values (not to be confused with the DL-based IM features in MSBooster) as features for Percolator as well (**Supplementary Fig 11a-b, Supplementary Data 1**). While 1/K0 by itself did not improve over only using spectral and RT features, the combination of charge and 1/K0 led to a 4.4% increase over baseline peptide identifications without MSBooster. Simply adding charge and 1/K0

values provides greater boosts compared to our hand-crafted IM feature, but the improvement is still minimal.

Supplementary References

1 Toprak, U. H. *et al.* Conserved Peptide Fragmentation as a Benchmarking Tool for Mass Spectrometers and a Discriminating Feature for Targeted Proteomics. *Molecular & Cellular Proteomics : MCP* **13**, 2056-2056 (2014).

2 Li, Y. *et al.* Spectral entropy outperforms MS/MS dot product similarity for small-molecule compound identification. *Nature Methods* **18**, 1524-1531 (2021).
