# Supplementary material for: MSBooster: improving peptide identification rates using deep learning-based features
Source: Nat Commun. 2023 Jul 27;14:4539. doi: 10.1038/s41467-023-40129-9 (PMC10374903; doi:10.1038/s41467-023-40129-9)
Supplement: Supplementary file 3 — Reporting Summary [file 41467_2023_40129_MOESM3_ESM.pdf]

nature portfolio

Corresponding author(s): Fengchao Yu, Alexey I. Nesvizhskii

Last updated by author(s): June 14, 2023

Reporting Summary

Nature Portfolio wishes to improve the reproducibility of the work that we publish. This form provides structure for consistency and transparency in reporting. For further information on Nature Portfolio policies, see our Editorial Policies and the Editorial Policy Checklist.

Statistics

For all statistical analyses, confirm that the following items are present in the figure legend, table legend, main text, or Methods section.

n/a	Confirmed	
☐	☒	The exact sample size (*n*) for each experimental group/condition, given as a discrete number and unit of measurement
☐	☒	A statement on whether measurements were taken from distinct samples or whether the same sample was measured repeatedly
☐	☒	The statistical test(s) used AND whether they are one- or two-sided<br>*Only common tests should be described solely by name; describe more complex techniques in the Methods section.*
☐	☒	A description of all covariates tested
☒	☐	A description of any assumptions or corrections, such as tests of normality and adjustment for multiple comparisons
☐	☒	A full description of the statistical parameters including central tendency (e.g. means) or other basic estimates (e.g. regression coefficient) AND variation (e.g. standard deviation) or associated estimates of uncertainty (e.g. confidence intervals)
☐	☒	For null hypothesis testing, the test statistic (e.g. *F*, *t*, *r*) with confidence intervals, effect sizes, degrees of freedom and *P* value noted<br>*Give P values as exact values whenever suitable.*
☒	☐	For Bayesian analysis, information on the choice of priors and Markov chain Monte Carlo settings
☒	☐	For hierarchical and complex designs, identification of the appropriate level for tests and full reporting of outcomes
☒	☐	Estimates of effect sizes (e.g. Cohen's *d*, Pearson's *r*), indicating how they were calculated

Our web collection on statistics for biologists contains articles on many of the points above.

Software and code

Policy information about availability of computer code

Data collection	Data was downloaded from ProteomeXchange and MassIVE via the FTP sites.
Data analysis	Venn diagrams were created at https://www.meta-chart.com/venn#/display or with Matplotlib.<br>Prosit predictions from https://www.proteomicsdb.org/prosit/ were generated with the “Prosit_2020_intensity_hcd” and “Prosit_2019_irt” models.<br>HLA peptide clustering was performed with GibbsCluster 2.0 (https://services.healthtech.dtu.dk/ service.php?GibbsCluster-2.0).<br>HLA binding prediction was performed with the NetMHC software (https://services.healthtech.dtu.dk/ service.php?NetMHC-4.0; https://services.healthtech.dtu.dk/services/NetMHCpan-4.1/)<br><br>Database searches were performed using MSFragger v3.4 in FragPipe v17.2 with Philosopher v4.1.1. For neoantigen detection, MSFragger v3.7, FragPipe v19.2, and Philosopher v5.0.0 were used for visualization of spectra with FragPipe-PDV viewer.<br><br>MSBooster code is available freely and as open source at https://github.com/Nesvilab/MSBooster.<br><br>Figure generation and data analysis was performed in an Anaconda environment with the following packages:<br>anaconda 2020.02<br>conda 4.8.2<br>joypy 0.2.6<br>jupyterlab 1.2.6<br>matplotlib 3.1.3<br>matplotlib-venn 0.11.7

numpy 1.18.1
pandas 1.3.0
python 3.7.6
scipy 1.4.1
seaborn 0.10.0

For manuscripts utilizing custom algorithms or software that are central to the research but not yet described in published literature, software must be made available to editors and reviewers. We strongly encourage code deposition in a community repository (e.g. GitHub). See the Nature Portfolio guidelines for submitting code & software for further information.

Data

Policy information about availability of data

All manuscripts must include a data availability statement. This statement should provide the following information, where applicable:

- Accession codes, unique identifiers, or web links for publicly available datasets
- A description of any restrictions on data availability
- For clinical datasets or third party data, please ensure that the statement adheres to our policy

Data availability
MS/MS datasets used in this study can be found at the ProteomeXchange Consortium and the PRIDE partner repository [89] or at the MassIVE repository with the following accession codes:
• HeLa timsTOF DDA PXD010012 [70]: https://proteomecentral.proteomexchange.org/cgi/GetDataset?ID=PXD010012
• HLA peptidome MSV000087743 [57]: https://massive.ucsd.edu/ProteoSAFe/dataset.jsp?task=a1638beae5d04267a99f92c550c60b34
• Melanoma neoantigen PXD004894 [65]: https://proteomecentral.proteomexchange.org/cgi/GetDataset?ID=PXD004894
• Melanoma DIA PXD022992 [66]: https://proteomecentral.proteomexchange.org/cgi/GetDataset?ID=PXD022992
• Single cell nanoPOTS MSV000085230 [67]: https://massive.ucsd.edu/ProteoSAFe/dataset.jsp?task=3013fc11dc4e4b6dae49a244d92854a7
• Single cell DISCO PXD019958 [45]: https://proteomecentral.proteomexchange.org/cgi/GetDataset?ID=PXD019958
• Secretome PXD026436 [69]: https://proteomecentral.proteomexchange.org/cgi/GetDataset?ID=PXD026436
All MSFragger produced pepXML, MSBooster-annotated pin, and fasta files are available at https://doi.org/10.5281/zenodo.8034585 and https://doi.org/10.5281/zenodo.7843558. Data used to generate the main and supplementary figures are provided in the Source Data file.

MHC allele binding motifs were acquired at the Immune Epitope Database (https://www.iedb.org/).

Research involving human participants, their data, or biological material

Policy information about studies with human participants or human data. See also policy information about sex, gender (identity/presentation), and sexual orientation and race, ethnicity and racism.

Reporting on sex and gender	Not applicable
Reporting on race, ethnicity, or other socially relevant groupings	Not applicable
Population characteristics	Not applicable
Recruitment	Not applicable
Ethics oversight	Not applicable

Note that full information on the approval of the study protocol must also be provided in the manuscript.

Field-specific reporting

Please select the one below that is the best fit for your research. If you are not sure, read the appropriate sections before making your selection.

☒ Life sciences ☐ Behavioural & social sciences ☐ Ecological, evolutionary & environmental sciences

For a reference copy of the document with all sections, see nature.com/documents/nr-reporting-summary-flat.pdf

Life sciences study design

All studies must disclose on these points even when the disclosure is negative.

Sample size	Mass spectrometry data was acquired from 7 prior studies for reanalysis. We believe these datasets span a relevant range of analyses for different search spaces (large nonspecific immunopeptidome databases to smaller in-silico human digests) and differing numbers of files (1 file analyzed for secretome data to multiple biological replicates for the single cell data).
Data exclusions	No data excluded.

Replication	Two separate single cell datasets and two separate HLA datasets were analyzed. Improvements in the number of identified peptides were evident in both, although various differences in experimental design make it difficult to explain why there are performance differences or even if they are meaningful differences.
Randomization	Randomization was performed by the labs that generated the data we used from public repositories. It was not possible to perform randomization when running our analyses, as the files from each study were, appropriately, analyzed together for each study.
Blinding	The objective of this study was to demonstrate our software's ability to identify more peptides and proteins. In making these comparisons, it was necessary to analyze different data files by their condition, such as number of cells or which files were matched to a specific melanoma patient. As such, blinding is not relevant here.

Reporting for specific materials, systems and methods

We require information from authors about some types of materials, experimental systems and methods used in many studies. Here, indicate whether each material, system or method listed is relevant to your study. If you are not sure if a list item applies to your research, read the appropriate section before selecting a response.

Materials & experimental systems

n/a	Involved in the study
☒	☐ Antibodies
☒	☐ Eukaryotic cell lines
☒	☐ Palaeontology and archaeology
☒	☐ Animals and other organisms
☒	☐ Clinical data
☒	☐ Dual use research of concern
☒	☐ Plants

Methods

n/a	Involved in the study
☒	☐ ChIP-seq
☒	☐ Flow cytometry
☒	☐ MRI-based neuroimaging
