# Supplementary material for: MSBooster: improving peptide identification rates using deep learning-based features
Source: Nat Commun. 2023 Jul 27;14:4539. doi: 10.1038/s41467-023-40129-9 (PMC10374903; doi:10.1038/s41467-023-40129-9)
Supplement: Supplementary file 4 — Description of Additional Supplementary Files Document [file 41467_2023_40129_MOESM4_ESM.pdf]

Description of Additional Supplementary Files

File Name: Supplementary Data 1

Description: Statistics for identified peptides/proteins when using different MSBooster features

File Name: Supplementary Data 2

Description: Reported canonical and non-canonical HLA peptides for Mel15 patient
